# Experimental evidence that EPA and DHA are dietary requirements in a migratory shorebird, but they do not affect muscle oxidative capacity

**DOI:** 10.1242/jeb.246105

**Published:** 2024-02-21

**Authors:** Morag F. Dick, Keith A. Hobson, Christopher G. Guglielmo

**Affiliations:** Department of Biology, Centre for Animals on the Move, Advanced Facility for Avian Research, Western University, London, ON, Canada, N6A 5B7

**Keywords:** Migration, Shorebird, Flight muscle, n-3 PUFA, Fatty acid nutrition

## Abstract

Dietary n-3 long chain polyunsaturated fatty acids (LCPUFAs) are hypothesized to be natural doping agents in migratory shorebirds, enabling prolonged flight by increasing membrane fluidity and oxidative capacity of the flight muscles. Animals can obtain n-3 LCPUFAs from the diet or by conversion of dietary α-linolenic acid, 18:3 n-3. However, the capacity to meet n-3 LCPUFA requirements from 18:3 n-3 varies among species. Direct tests of muscle oxidative enhancement and fatty acid conversion capacity are lacking in marine shorebirds that evolved eating diets rich in n-3 LCPUFAs. We tested whether the presence and type of dietary fatty acids influence the fatty acid composition and flight muscle oxidative capacity in western sandpipers (*Calidris mauri*). Sandpipers were fed diets low in n-3 PUFAs, high in 18:3 n-3, or high in n-3 LCPUFAs. Dietary fatty acid composition was reflected in multiple tissues, and low intake of n-3 LCPUFAs decreased the abundance of these fatty acids in all tissues, even with a high intake of 18:3 n-3. This suggests that 18:3 n-3 cannot replace n-3 LCPUFAs, and dietary n-3 LCPUFAs are required for sandpipers. Flight muscle indicators of enzymatic oxidative capacity and regulators of lipid metabolism did not change. However, the n-3 LCPUFA diet was associated with increased FAT/CD36 mRNA expression, potentially benefitting fatty acid transport during flight. Our study suggests that flight muscle lipid oxidation is not strongly influenced by n-3 PUFA intake. The type of dietary n-3 PUFA strongly influences the abundance of n-3 LCPUFAs in the body and could still impact whole-animal performance.

## Teaser

**Summary:** Dietary manipulation of n-3 PUFAs suggests that western sandpipers have a dietary requirement for EPA and DHA. However, n-3 PUFAs do not increase flight muscle oxidative capacity.

## INTRODUCTION

The ability of animals to perform during energetically demanding life-history stages may depend on the availability of key dietary factors and essential nutrients. For birds, a critical step in preparing for migration is increasing fat stores and the capacity to oxidize fatty acids to fuel migratory flight (Guglielmo, 2018). Although fat is the main fuel for migratory flight, fat quality can also influence an animal's physiology and modulate flight performance through other mechanisms (Price, 2010). A major focus of nutritional research in migratory birds has been on the source and importance of dietary polyunsaturated fatty acids (PUFAs), which are readily incorporated and reflected in tissue PUFA composition (Guglielmo, 2018; Pierce and McWilliams, 2014; Price, 2010). The dominant type of dietary PUFA depends on an animal's foraging environment, with n-3 long chain PUFAs (LCPUFAs), eicosapentaenoic acid (20:5 n-3, EPA) and docosahexaenoic acid (22:6 n-3; DHA), being found primarily in marine and aquatic environments (Colombo et al., 2017; Twining et al., 2021). In comparison, the fundamental precursor to EPA and DHA, α-linolenic acid (18:3 n-3) and n-6 PUFAs, such as linoleic acid (18:2 n-6), are often associated with terrestrial ecosystems (Twining et al., 2021). The types of dietary PUFA consumed may have different effects on migratory performance, and these effects could be species specific. For example, the n-6 PUFA 18:2 n-6 reduced flight energy costs in songbirds (Carter et al., 2020; McWilliams et al., 2020), whereas, for marine-associated shorebirds, n-3 LCPUFAs have been proposed to be important for migration performance (Weber, 2009).

The natural doping hypothesis posits that dietary n-3 LCPUFAs may influence fatty acid metabolism and prime the flight muscles for endurance flight through a variety of mechanisms (Price, 2010; Weber, 2009). The first mechanism is n-3 LCPUFAs serving as strong ligands for peroxisome proliferator activator receptors (PPAR), which are major regulators of lipid metabolism, and it is predicted that increasing n-3 LCPUFAs would upregulate the expression of genes involved in fatty acid metabolism, leading to increased muscle oxidative capacity (Weber, 2009). The second proposed mechanism is n-3 LCPUFA incorporation into the membranes of the flight muscle, thereby altering the activity of membrane-bound proteins in cellular and organelle membranes (Weber, 2009). Elevated activity of key oxidative enzymes in association with n-3 LCPUFA abundance in the membranes in refuelling migratory semipalmated sandpipers (*Calidris pusilla*) and sedentary bobwhite quail (*Colinus virginianus*) supports the hypothesis that n-3 LCPUFAs are important for some migrating birds (Maillet and Weber, 2006, 2007; Nagahuedi et al., 2009). In contrast to these findings, controlled diet studies using migratory songbirds have found n-3 LCPUFAs had no effect, or even decreased oxidative capacity, and showed no improvement in measures of whole-animal performance (Dick and Guglielmo, 2019; Price and Guglielmo, 2009). However, as most songbirds naturally consume terrestrial diets dominated by n-6 PUFAs, it may be that their performance is enhanced by dietary n-6 PUFAs, whereas bird species that have evolved to consume marine diets rich in n-3 LCPUFAs, such as shorebirds, need and benefit from these fatty acids (Young et al., 2021).

One of the underlying questions about n-3 PUFAs and their effects on animals is whether the type of PUFA consumed matters. Overall, n-3 PUFAs are a group of essential nutrients. However, the ability to elongate and desaturate 18:3 n-3 to the LCPUFA (EPA and DHA) may influence what type of n-3 PUFA needs to be consumed, and whether EPA and DHA are specifically required in the diet (Twining et al., 2021). Species that evolved on diets with a source of n-3 LCPUFAs, such as domestic cats (*Felis catus*), have functionally lost the hepatic enzymatic capacity to elongate and desaturate 18:3 n-3, and therefore require a dietary source of EPA and DHA (Castro et al., 2012; Rivers et al., 1975). In effect, cats rely on the conversion ability of their prey (often herbivores). A similar pattern is found in salmonid fish, where marine species with consistent availability of high n-3 LCPUFAs in the diet have poor conversion ability, whereas those from freshwater systems retain a greater ability to produce EPA and DHA from 18:3 n-3 (Glencross, 2009). The capability to elongate and desaturate 18:3 n-3 can be observed by measuring the incorporation of dietary short chain PUFAs into LCPUFAs in the tissues (Ben-Hamo et al., 2011; Dick and Guglielmo, 2019; Lopez-Ferrer et al., 2001; Petzinger et al., 2014). The conversion capacity may be limited in tree swallows (*Tachycineta bicolor*), suggesting that capacity may vary even among terrestrial avian species, and should be considered when assessing dietary requirements (Twining et al., 2018). Diversity in desaturation and elongation capacity is especially important for the understudied marine and semi-aquatic species because it is predicted that global climate change will decrease the natural availability of n-3 LCPUFAs (Colombo et al., 2017). Therefore, understanding whether the type of dietary n-3 PUFA consumed influences physiological processes is also needed to inform how environmental factors and diet may influence migratory performance.

Here, we had two main objectives. The first was to establish how manipulating the type and presence of dietary n-3 PUFAs influences the fatty acid composition of key tissues of a marine-associated shorebird, the western sandpiper (*Calidris mauri*). We predicted that a limited conversion capacity of 18:3 n-3 to n-3 LCPUFAs would lead to a decrease in tissue abundance of n-3 LCPUFAs. Second, we tested whether the type and presence of n-3 PUFAs influenced muscle lipid transport and oxidative capacity, as proposed by the natural doping hypothesis. We predicted that if all n-3 PUFAs have similar PPAR binding affinities, then increasing n-3 PUFA intake would increase oxidative capacity through changes in muscle transporters and enzyme activity regardless of n-3 PUFA type.

## MATERIALS AND METHODS

### Animals and experimental design

Juvenile western sandpipers, *Calidris mauri* (Cabanis 1857), were captured near Roberts Bank in Delta, BC, Canada, in August 2020. The birds were housed for ∼36 h before being air shipped directly to Toronto, ON, Canada, and then transported by vehicle to the Advanced Facility for Avian Research at the University of Western Ontario, London, ON, Canada. All animal capture and procedures were approved and under a protocol from the University of Western Ontario Animal Care Committee (2018-092) and permitted by the Canadian Wildlife Service (SC-BC-2020-0021).

The birds were housed in groups in specialized shorebird holding rooms (2.4×3.7 m) which were environmentally enriched with trays of sand, a variety of soft substrates to walk on, and a running stream and pool. The light cycle was maintained at 12 h:12 h light:dark, with a temperature of ∼21°C throughout the entire experiment. Fresh food and water were provided *ad libitum*. The birds were acclimated to a base diet of ground, water-soaked low-fat dog kibble (President's Choice Nutrition First Senior Dog Food; 9% fat on a dry matter basis) with canola oil added to increase the fat content of the diet (16% fat on a dry matter basis). During the experimental diet phase, the types of oils were modified.

In late October 2020, the birds were randomized into groups of 8 and fed one of three experimental diets differing in fatty acid profile (36.1% dog food, 63.12% water, 0.78% experimental oil blend; Table 1). The oil blends were formulated to maximize the differences in n-3 PUFAs and minimize differences in other fatty acids. To best approximate the composition of the invertebrates and biofilm consumed by western sandpipers on the mudflats (Quinn et al., 2017; Schnurr et al., 2019, 2020), we sourced a commercial algal oil (DSM Life's Omega 60 algal oil). This algal oil, along with coconut oil and olive oil, was added to the dog food to create a diet rich in long-chain n-3 PUFAs (LCPUFA diet; Table 1). A blend of flaxseed oil and coconut oil was used to generate a diet rich in α-linolenic acid (18:3 n-3; ALA diet), a precursor fatty acid of long-chain n-3 PUFAs including EPA and DHA. Finally, a diet low in n-3 PUFAs and enriched in monounsaturated fatty acids (MUFA diet) was made using olive oil and coconut oil.

**Table 1.**
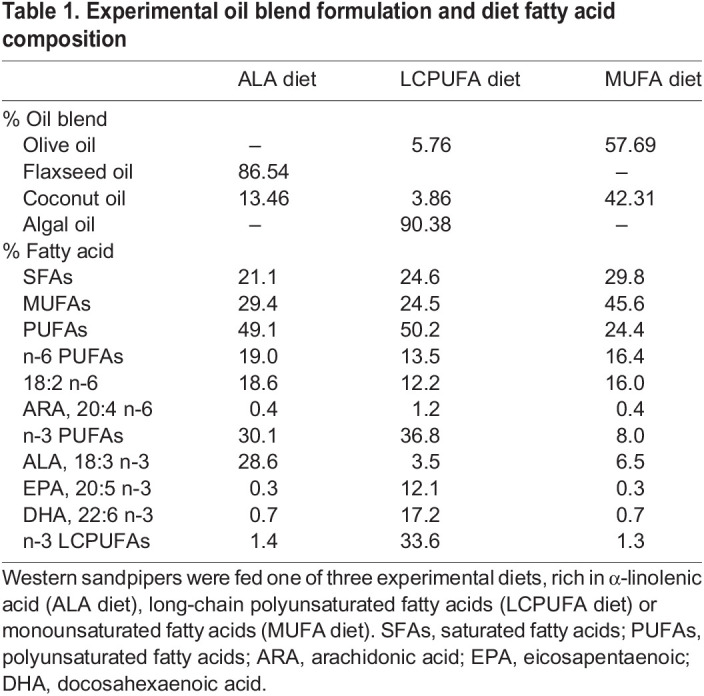
Experimental oil blend formulation and diet fatty acid composition

Birds were slowly switched onto their experimental diets by mixing the new diet with the base diet (day 1: 25%, day 2 and 3: 50%, and day 4: 100% experimental diet). All birds transitioned well to their new diets, and we observed no diet-related health issues throughout the study. Each week, the birds were weighed, and an overall health assessment was done. At the end of the fourth week, the birds were weighed, and their body composition (lean and fat mass) was measured using quantitative magnetic resonance (Guglielmo et al., 2011). The birds were then humanely euthanized under isoflurane anaesthesia, and blood was collected and centrifuged for plasma and blood cell fractions. The flight muscle, liver, adipose and brain were collected for further analysis. Samples were flash frozen in liquid nitrogen and stored at −80°C for later analysis.

### Fatty acid analysis

Fatty acid composition (mass %) of plasma, blood cells, adipose, muscle, liver and brain were analysed following Dick and Guglielmo (2019). Briefly, total lipids were extracted from 12 µl each of the plasma and blood cell fractions of blood, 10–15 mg of adipose and brain, and 30–50 mg for liver and flight muscle. Samples were homogenized (vortexed for plasma) in chloroform–methanol–0.1% butylated hydroxytoluene (2:1:0.003 v/v/w) and then centrifuged prior to the addition of 0.25% potassium chloride, and incubated at 70°C for 5 min. The bottom layer organic (lipid) layer was dried under a nitrogen stream. In the case of the liver and flight muscle tissues, we further separated the total lipids into neutral and polar lipid fractions by column chromatography using Supelclean solid phase extraction columns (Sigma-Aldrich, Oakville, ON, Canada). Briefly, neutral lipids were eluted with chloroform:isopropanol (2:1 v/v), followed by the elution of non-esterified fatty acids (NEFAs) with isopropyl ether:acetic acid (49:1 v/v), and finally the phospholipid fraction was eluted with methanol. We did not separate the lipid fractions of adipose because the fatty acid profile of the total lipids is almost entirely reflective of the neutral lipid fraction (Klaiman et al., 2009). We also analysed fatty acid composition of brain total lipids to maintain continuity with previously published data in birds (Lamarre et al., 2021; Twining et al., 2016).

Fatty acid methyl esters (FAMEs) were generated using 0.5 mol l^−1^ methanolic-HCl (Sigma-Aldrich) for 30 min at 90°C. Afterward, purified water was added, the FAMEs were extracted three times with hexane, then dried under a stream of N_2_ gas and resuspended in 60 µl hexane. We then separated the FAMEs using gas chromatography (6890N gas chromatograph, Agilent Technologies, Santa Clara, CA, USA) with a flame ionization detector and DB-23 column (Agilent Technologies) with helium as the carrier gas. The temperature program was set to 80°C for 2 min, followed by a 5°C min^−1^ ramp to 180°C, which was held for 5 min, and then a 1°C min^−1^ ramp to 200°C, and finishing with a 10°C min^−1^ ramp to 240°C, which was held for 3 min. Fatty acid retention times were determined using commercial standards (Supelco 37 mix and Supelco PUFA No. 3 Menhaden Oil, Sigma-Aldrich). The fatty acid mass percentage was calculated for each fatty acid by dividing the fatty acid peak area by the total peak area of all identified fatty acids.

### Flight muscle fatty oxidative capacity

We measured the relative mRNA abundance of major regulators of lipid metabolism (PPARα, PPARβ and PPARγ, PGC1α), and membrane and cytosolic fatty acid transporters (FAT/CD36 and FABP3) in the flight muscle. RNA isolation and DNAse treatment were carried out using ∼30 mg of flight muscle tissue with a GENEzol TriRNA Pure kit (Geneaid, New Taipei City, Taiwan) followed by cDNA synthesis using a High-Capacity RNA-to-cDNA kit (Applied Biosystems, Burlington, ON, Canada), with the resulting cDNA diluted 100-fold with water. Quantitative real-time PCR (qPCR) was performed using the Quantstudio™ 3 Real-Time PCR System (Thermo Fisher Scientific), with the following PCR reaction conditions: 2 µl diluted cDNA, 6 µl master mix (SensiFast SYBR and Fluorescein Mix, Bioline, Taunton, MA, USA) and 0.33 mol l^−1^ of the primer pairs (Table 2), in a total reaction volume of 12 µl. The PCR cycling conditions were as follows: 95°C for 10 min, followed by 45 cycles of 95°C for 20 s, 59°C for 20 s, and 72°C for 10 s. All samples were run in triplicate. The arithmetic mean of *GAPDH* and *β-actin* was used as the housekeeping gene. Relative mRNA expression was calculated using the 2^ΔΔ^CT method, and the arithmetic mean expression of the MUFA group was set to 1.

**Table 2.**
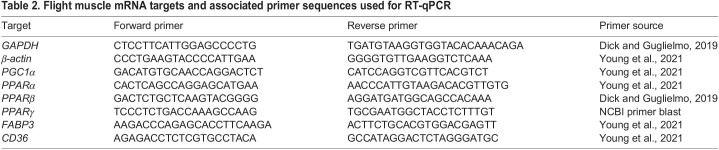
Flight muscle mRNA targets and associated primer sequences used for RT-qPCR

Flight muscle maximum activity of carnitine palmitoyl transferase (CPT; EC 2.3.1.21), citrate synthase (CS; EC 2.3.3.1), 3-hydroxy acyl-CoA dehydrogenase (HOAD; EC 1.1.1.35) and lactate dehydrogenase (LDH; EC 1.1.1.27) were measured following Price et al. (2010). Briefly, approximately 50 mg of flight muscle was homogenized with 9 volumes of buffer (20 mmol l^−1^ Na_2_HPO_4_, 0.5 mmol l^−1^ EDTA, 0.2% defatted BSA, 50% glycerol, 0.1% Triton X-100 and aprotinin at 50 µg ml^−1^) and the homogenates diluted 10-fold with the buffer and stored at −80°C until analysis. Enzyme activity was measured at 39°C in 1 ml total volume on a Cary 100 Bio Spectrophotometer (Varian, Palo Alto, CA, USA) and calculated from the change in absorbance at 412 nm for CS and CPT and 340 nm for HOAD and LDH. CPT was assayed in 50 mmol l^−1^ Tris buffer (pH 8), with 5 mmol l^−1^ carnitine, 0.15 mmol l^−1^ DTNB and 0.035 mmol l^−1^ palmitoyl CoA. CS was measured using 50 mmol l^−1^ Tris buffer (pH 8), 0.5 mmol l^−1^ oxaloacetic acid, 0.15 mmol l^−1^ DTNB and 0.3 mmol l^−1^ acetyl CoA. HOAD was measured in a 50 mmol l^−1^ imidazole buffer (pH 7.4), with 0.2 nmol l^−1^ NADH, 1 mmol l^−1^ EDTA and 0.1 mmol l^−1^ acetoacetyl CoA. LDH was assayed in the same imidazole buffer, with 8 mmol l^−1^ pyruvate, 0.3 mmol l^−1^ NADH and 5 mmol l^−1^ DTT.

### Statistics

All statistical analyses were performed using R v.1.1463 (http://www.R-project.org/) and RStudio (https://posit.co/products/open-source/rstudio/) software. Weekly body mass was analysed using a linear mixed effects model to test the main effects and interaction of diet and week, with bird ID included as a random effect. Final body mass and other body composition data were analysed using a linear model testing for the effect of diet. Significant main effects of diet were followed up with Tukey's *post hoc* tests.

To analyse the fatty acid data, we first did a MANOVA to test the main effects of diet and tissue type on fatty acid composition. This was followed with a compositional analysis of the fatty acid profiles done using easyCoDa (https://CRAN.R-project.org/package=easyCODA), a method developed to overcome the issues of using compositional data for multivariate and principal component analysis (PCA; Graziano et al., 2020). easyCoDa uses a central-log ratio (CLR) transformation of the fatty acid values for the PCA. Only fatty acids that comprised greater than 1% of the total in all groups were used in the PCA. To test the effect of diet within the tissues, MANOVA was done on each tissue type, testing the effect of diet on fatty acid composition, and if significant followed up with linear models on each fatty acid. Fatty acid composition for each tissue was analysed for fatty acids that comprised greater than 1% of total or were considered key n-3 or n-6 PUFAs. To determine whether the fatty acid composition of key tissues could be measured non-invasively by taking blood samples, we used linear models to test for relationships between key PUFAs in the total lipid extracts of plasma and blood cells, adipose and brain, and phospholipid lipid fractions of muscle and liver.

Muscle enzyme activity was analysed with linear models testing the effect of diet, where body mass was initially assessed as a covariate and removed from the model if not significant. Relative mRNA abundance was analysed in the same way but log-transformed before analysis. Significant effects of diet were followed up with Tukey's *post hoc* test. The significance level was set to *P*<0.05.

## RESULTS

### Body composition and mass

**Fig. 1.**
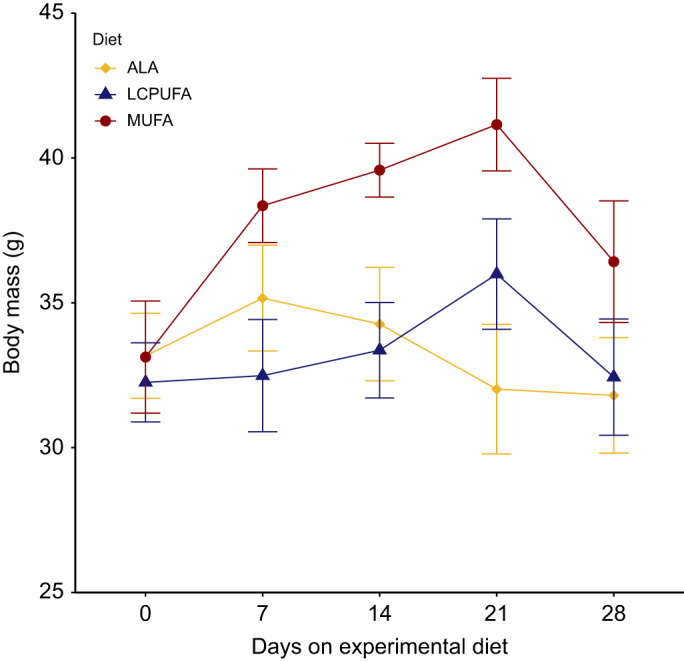
**Weekly body mass of western sandpipers during the 4** **week feeding trial.** Birds were fed one of three diets, rich in α-linolenic acid (ALA diet), long chain polyunsaturated fatty acids (LCPUFA diet) or monounsaturated fatty acids (MUFA diet). Values are means±s.e.m., *n*=8 per diet group.

Over the course of the feeding trial, diet and time had a significant main effect on body mass, with a trend for the interaction of the two effects (Diet: *F*_2,21_=4.2713, *P*=0.028; Time: *F*_4,84_=3.321, *P*=0.014; Diet×Time=*F*_8,84_=1.799, *P*=0.089). The MUFA diet group was heavier and body mass overall increased in weeks 2 and 3 compared with the start (Fig. 1). There were no differences in body mass between groups at the start or at the end of the trial. Additionally, we found no significant differences among diet treatments in body mass, or fat, lean, heart, liver or flight muscle mass (Table 3).

**Table 3.**
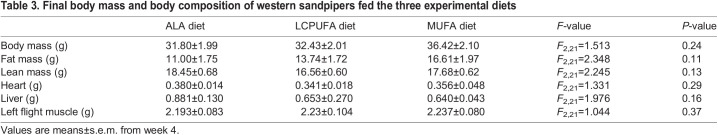
Final body mass and body composition of western sandpipers fed the three experimental diets

### Fatty acid composition

**Fig. 2.**
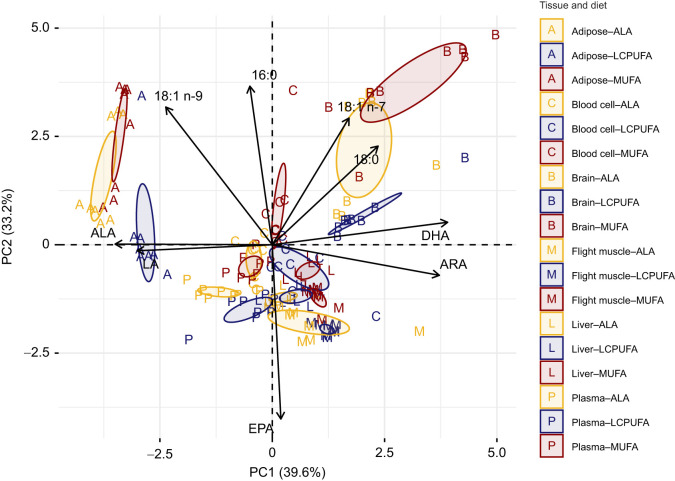
**Principal component analysis (PCA) biplot of the tissue fatty acid profiles of western sandpipers fed the three experimental diets.** Colours represent the different experimental diets, and the coloured uppercase letters represents each tissue. Ellipses represent the 95% confidence interval around the group mean for each diet and tissue combination (*n*=8 per diet group). See Table S4 for PC loadings.

Fatty acid composition varied with the experimental diets and tissue (MANOVA: Diet: *F*_18,238_=53.135, *P*<0.0001; Tissue: *F*_45,610_=39.907, *P*<0.0001; Diet×Tissue: *F*_90,1134_=5.092, *P*<0.0001). The CLR-transformed fatty acid profiles were used in the PCA analysis to categorize the tissues, with subclasses within tissues emerging according to diet in some cases (Fig. 2; Table S4). The principal component analysis separated out the diets and tissues with above-average contributions from DHA, 20:4 n-6, 18:3 n-3 and 18:2 n-6 for PC1 and EPA, 16:0, 18:1 n-9 and 18:1 n-7 for PC2. This resulted in adipose and brain tissues not overlapping with any other tissue in the multidimensional space, and within both of these tissues the LCPUFA diet group separated from both the ALA and MUFA diet groups. The flight muscle, liver, blood cell and plasma grouped more centrally together; however, for blood cell and plasma there was no diet overlap, and for liver and flight muscle the MUFA diet group did not overlap with the LCPUFA and ALA diet groups.

**Fig. 3.**
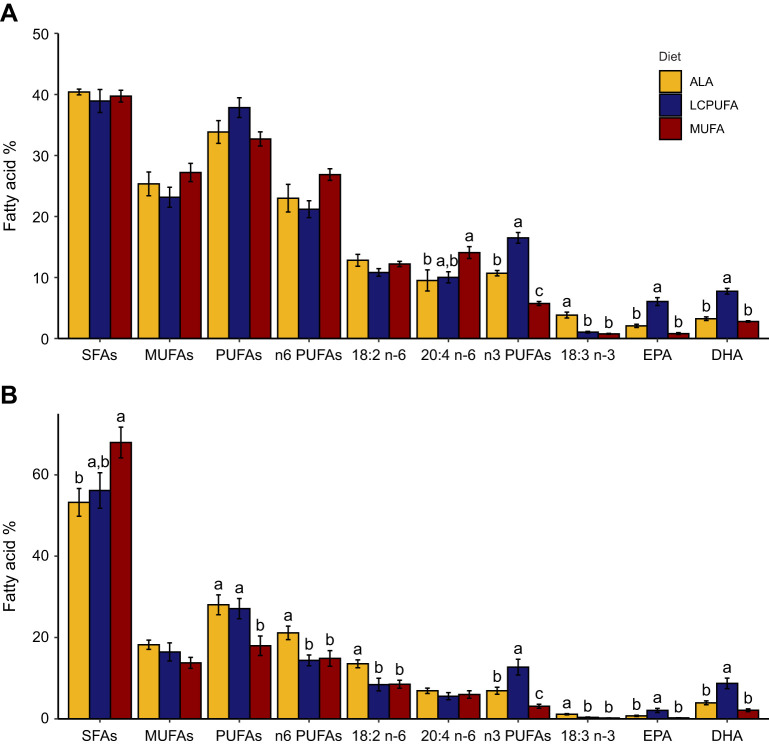
**Summary of fatty acid composition of the blood of western sandpipers fed the experimental diets.** (A) Plasma. (B) Blood cells. Fatty acids or fatty acid classes that have different letter groupings differ significantly (*P*<0.05, *n*=8 per diet group). SFAs, saturated fatty acids; PUFAs, polyunsaturated fatty acids. Detailed fatty acid composition is provided in Table S1.

**Fig. 4.**
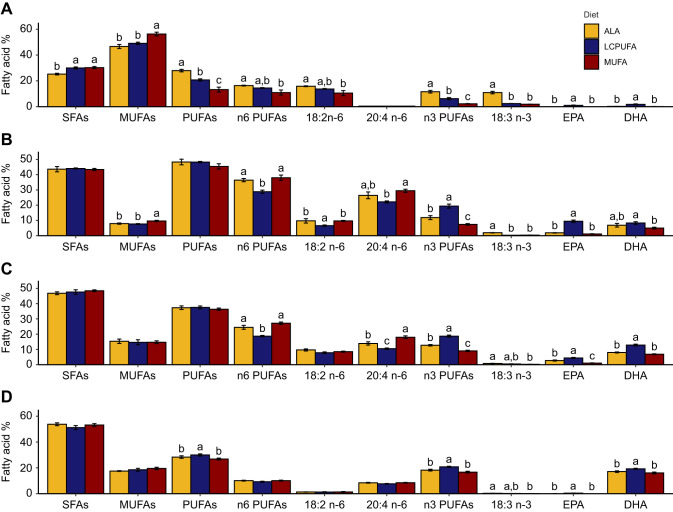
**Summary of tissue fatty acid composition of western sandpipers fed the experimental diets.** (A) Adipose, (B) flight muscle phospholipids, (C) liver phospholipids and (D) brain. Fatty acids or fatty acid classes that have different letter groupings differ significantly (*P*<0.05, *n*=8 per diet group). Detailed fatty acid compositions are provided in Tables S2 and S3.

The experimental diets overall influenced the plasma (MANOVA: *F*_18,28_=44.051, *P*<0.0001) and blood cell (MANOVA: *F*18,28=44.051, *P*<0.0001) fatty acid composition. For both these blood fractions, the highest total n-3 PUFA level was observed in the LCPUFA diet group because of higher EPA and DHA. In comparison, the ALA diet group had intermediate levels of n-3 PUFAs in plasma and blood cells due to an increased proportion of 18:3 n-3 (Fig. 3; statistics provided in Tables S1). Additionally, in the plasma, an increased abundance of 20:4 n-6 was observed in the MUFA diet group. The diets also significantly influenced adipose composition (MANOVA: *F*_18,28_=15.938, *P*<0.0001). Here, the MUFA diet group had the lowest total PUFA level, but a higher MUFA level, reflecting the diet. Furthermore, the ALA diet group had the highest total PUFA level due to a higher abundance of both 18:2 n-6 and 18:3 n-3 (Fig. 4A; statistics provided in Table S2). Flight muscle (MANOVA: *F*_18,28_=21.03, *P*<0.0001) and liver (MANOVA: *F*_18,28_=9.1062, *P*<0.0001) phospholipid composition followed a similar pattern, with higher EPA and DHA, and lower 20:4 n-6 in the membranes of birds fed the LCPUFA diet compared with the ALA and MUFA diets (Fig. 4B,C; statistics provided in Tables S2 and S3). Finally, the diets had an overall effect on brain fatty acid composition (MANOVA: *F*_18,28_=5.992, *P*<0.0001), with the largest difference being in the abundance of DHA, with lower proportions in both the ALA and MUFA diet groups (Fig. 4D; statistics provided in Table S3).

We also examined whether plasma or blood cell PUFAs (18:2 n-6, 18:3 n-3, 20:4 n-6, EPA and or DHA) composition could be used as an indicator of tissue composition (Fig. S1 and Table S5). For flight muscle phospholipid fatty acid composition, we found significant linear relationships with 20:4 n-6, 18:3 n-3 and EPA of plasma, but only 18:3n-3 and EPA of blood cells. For adipose, 18:3 n-3, EPA and DHA of both plasma and blood cells, and 18:2 n-6 of blood cells were linearly related with adipose fatty acid composition. Liver phospholipid composition was linearly related with all key plasma PUFA proportions, but only linearly related with blood cell proportions of 18:3 n-3, EPA and DHA. Finally, brain fatty acid proportions of the n-3 PUFAs were linearly related to plasma and blood cell fatty acid composition.

### Flight muscle oxidative capacity

**Fig. 5.**
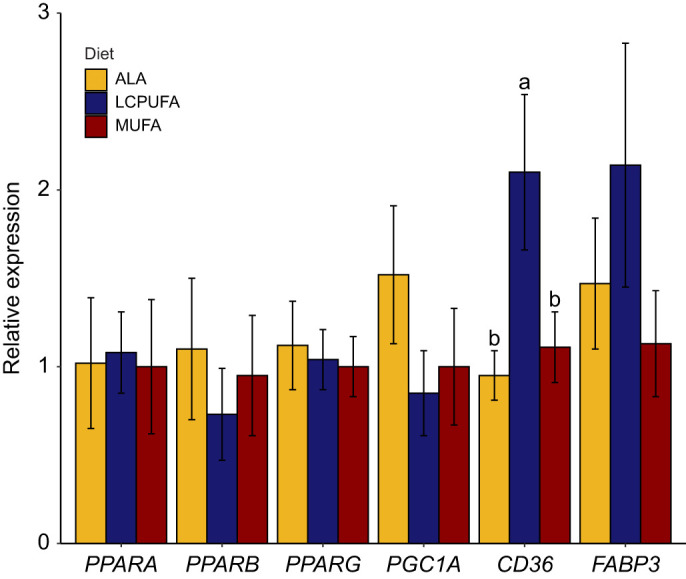
**Flight muscle relative mRNA abundance of key regulators of lipid metabolism and fatty acid transporters in western sandpipers fed the experimental diets.** Genes that have different letter groupings differ significantly (*P*<0.05, *n*=8 per diet group).

**Fig. 6.**
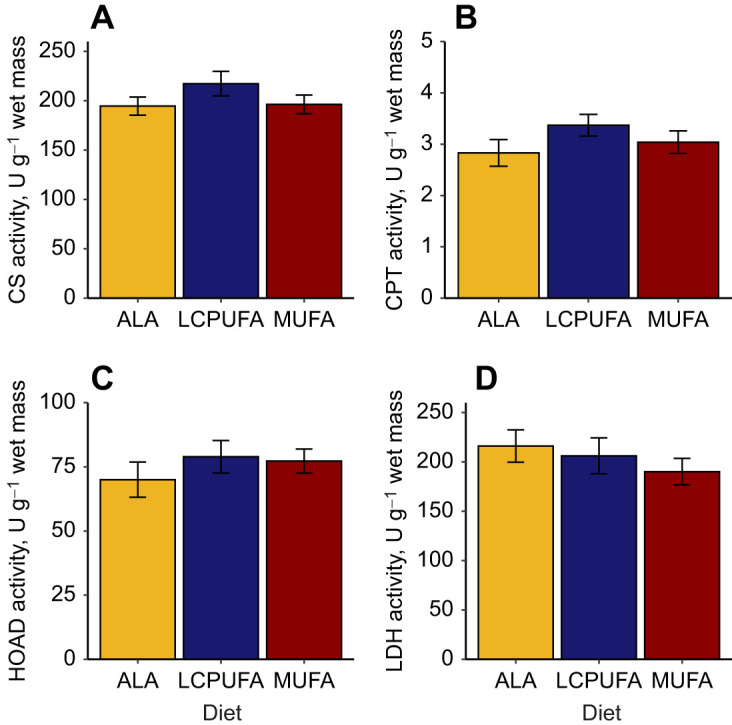
**Flight muscle oxidative enzyme activity of western sandpipers fed the experimental diets.** (A) Citrate synthase (CS), (B) carnitine palmitoyl transferase (CPT), (C) 3-hydroxyacyl-CoA dehydrogenase (HOAD) and (D) lactate dehydrogenase (LDH). There were no statistically significant differences (*n*=8 per diet group).

We measured the relative expression of key regulators of lipid metabolism (Fig. 5). No significant differences in the relative expression of *PPARα* (*F*_2,21_=0.74, *P*=0.49), *PPARβ* (*F*_2,21_=0.85, *P*=0.44), *PPARγ* (*F*_2,21_=0.47, *P*=0.63) or *PGC1α* (*F*_2,21_=0.27, *P*=0.76) were observed. There was a significant increase in the relative expression of fatty acid translocase *CD36* (*F*_2,21_=5.33, *P*=0.014) in the LCPUFA diet group, but not in the cytosolic fatty acid transporter *FABP3* (*F*_2,21_=1.15, *P* =0.34). At the enzyme level, CS activity did not significantly differ among diet groups (Fig. 6; *F*_2,21_=1.45, *P*=0.26), nor did we detect any differences in the other oxidative enzymes HOAD (*F*_2,21_=0.61, *P*=0.55) and CPT (*F*_2,21_=1.39, *P*=0.27), or the anaerobic enzyme LDH (*F*_2,21_=0.66, *P*=0.53).

## DISCUSSION

We tested how the type and presence of n-3 PUFAs influenced tissue fatty acid composition and flight muscle oxidative capacity in western sandpipers. We found that diet significantly influenced the fatty acid composition of the various tissues and that the type of n-3 PUFA consumed influenced the abundance of EPA and DHA in the tissues. However, there was very limited evidence for increased lipid transport capacity with the intake of n-3 PUFAs, and no effect on muscle oxidative capacity.

### Impact of diet on fatty acid composition

The type and presence of fatty acids, especially PUFAs, influence the composition of fatty acids in a bird's body, and some tissues, such as adipose, respond rapidly to dietary changes in a dose-dependent manner (Carter et al., 2020; Egeler et al., 2003; McWilliams et al., 2020; Nagahuedi et al., 2009; Neijat et al., 2017; Price and Guglielmo, 2009). As anticipated, birds consuming the n-3 LCPUFA diet had the highest levels of EPA and DHA in their tissues and maintained a similar flight muscle fatty acid composition to wild western sandpipers (Guglielmo et al., 2002). Sandpipers fed a diet low in n-3 LCPUFAs (ALA and MUFA diet groups) increased the proportion of 18:2 n-6 and 20:4 n-6 in the liver and flight muscle phospholipids, and this is a similar pattern to that found in songbird flight muscles (Dick and Guglielmo, 2019; Price and Guglielmo, 2009). The current study also included brain fatty acid composition, where low dietary n-3 LCPUFA was reflected in the brain by a decrease in the proportion of DHA, and was not compensated for with an increase in other PUFAs. DHA is important for brain health (Sun et al., 2018), and decreasing DHA could negatively impact cognition as suggested in fledging ring billed gulls (*Larus delawarensis*; Lamarre et al., 2021) The biological effect at the adult stage may require a greater difference than observed in the current study or be sub-clinical compared with young birds.

It is important to note that a high intake of 18:3 n-3 in the ALA diet did not result in similar proportions of n-3 LCPUFAs to those in the LCPUFA diet group. Although an animal may have the capacity to elongate and desaturate 18:3 n-3 to n-3 LCPUFAs, the capacity for this conversion may potentially not be sufficient to meet the animal's nutritional requirements (Twining et al., 2018). In the current study, we observed potential evidence of insufficient capacity to convert 18:3 n-3 to EPA and DHA in western sandpipers. After the 4 week feeding trial, birds consuming the ALA diet had intermediate or similar proportions of n-3 LCPUFAs to those on the MUFA diet in key tissues such as the brain and flight muscle. Furthermore, the proportion of 18:3 n-3 in the adipose was considerably higher than in birds on the other two diets. This pattern suggests that the birds were unable to convert enough 18:3 n-3 to EPA and DHA to maintain similar proportions to those found for birds on the LCPUFA diet, and this caused an accumulation of 18:3 n-3 in the adipose. Changes in the adipose composition that we observed are comparable to those found in chickens, where 18:3 n-3 was incorporated into the adipose in a dose-dependent manner compared with dietary EPA and DHA, which was not reflected as greatly in the adipose (Neijat et al., 2017). However, the lower n-3 LCPUFAs in key tissues and higher 18:3 n-3 in the adipose could potentially be due to the birds not needing to achieve the composition found in the wild, and is an artifact of diet and not nutrient requirements and/or low conversion capacity. Future studies should directly investigate the dynamics of the hepatic enzymes involved in fatty acid elongation and desaturation in western sandpipers and other marine birds and incorporate the potential effects of dietary history and seasonality on their activity.

The utility of using blood (whole-blood, plasma or blood cell) fatty acid composition to infer tissue fatty acid composition is appealing because of its low invasiveness and ease of sampling when studying free-living birds. In the current study, we tested for significant relationships between n-3 and n-6 PUFAs with blood cell and plasma fatty acid composition to provide insight into the suitability and potential limitations of blood markers. Overall, we found significant linear relationships with the n-3 PUFAs between the blood and tissue fatty acid compositions, validating that differences in dietary intake of n-3 PUFAs can be inferred from blood markers in western sandpipers. However, the current study was done under controlled conditions (consistent diet for 4 weeks and fasting prior to sampling), which cannot be assumed for wild birds. Plasma fatty acid composition is likely more dynamic and responsive to immediate changes from feeding state and diet as a result of its faster turnover rate than blood cells and other tissues (Pearson et al., 2003; Podlesak et al., 2005). Studies in wild populations could combine plasma and blood cell fatty acid profiles to develop a broader understanding of fatty acid intake, with plasma composition representing changes in the immediate days, and blood cell composition representing the net changes over weeks and a more stable marker of tissue composition.

In addition to blood sampling, adipose can be collected non-lethally from free-living animals, also allowing for fatty acid analysis (Morrissey and Swekla, 2023). However, its utility as an overall fatty acid biomarker may have limitations. The current study suggests that interpreting diet or other tissue fatty acid composition from adipose only may be limited because (1) the overall composition of adipose grouped away from the other tissues of interest in a multivariate analysis, (2) the proportion of n-3 PUFAs incorporated into adipose was dependent on the type of dietary n-3 PUFAs consumed (18:3 n-3 versus n-3 LCPUFAs), and (3) adipose contains a high proportion of saturated fatty acids (SFAs) and MUFAs which could be derived from de novo fatty acid synthesis and therefore influenced by the macronutrient composition of the diet (Budge et al., 2020). Assessing adipose fatty acid composition in conjunction with plasma and/or blood cell fatty acid composition in future studies will help to clarify the limitations and advantages of using adipose as a fatty acid marker.

### Influence of presence and type of n-3 PUFAs on lipid oxidation

The natural doping hypothesis posits that n-3 LCPUFAs can increase endurance flight capacity by increasing the abundance of ligands for PPAR, and by altering flight muscle membrane fluidity (Weber, 2009). Unlike in sedentary quail (Nagahuedi et al., 2009), we did not detect any significant differences in oxidative capacity at the enzyme level between diets, and no differences in PPAR mRNA expression. We did observe an effect on fatty acid transporters, with CD36 relative mRNA abundance being higher in the LCPUFA diet group, and FABP3 mRNA abundance tending in the same direction. However, without significant differences in oxidative enzymes among diets, dietary n-3 PUFAs likely do not greatly influence lipid metabolism at this level in western sandpipers. Previously, the role of dietary PUFAs in migratory birds focused on the class of PUFA (n-3 versus n-6 PUFAs: Dick and Guglielmo, 2019; Price and Guglielmo, 2009) or specifically on the role of the n-6 PUFA 18:2 n-6 (Carter et al., 2020; DeMoranville et al., 2021; Price et al., 2022). Generally, these studies found that dietary PUFAs have little to no influence on lipid oxidative capacity and that seasonal changes and/or exercise may have larger impacts on muscle physiology (Carter et al., 2021; Dick and Guglielmo, 2019; Price et al., 2022). Although EPA and DHA are potent ligands for PPAR, other fatty acids found in our diets can also be ligands, such as 18:3 n-3 and 18:2 n-6 (Hamilton et al., 2018; Mochizuki et al., 2006). Furthermore, increasing the availability of potent ligands does not necessarily increase the abundance of PPAR mRNA (Dick and Guglielmo, 2019; Nagahuedi et al., 2009), but can do so in isolated shorebird muscle *in vitro* (Young et al., 2021). Nor does increasing the availability of EPA and DHA necessarily alter lipid metabolism, and it can even decrease maximum oxidative enzyme activity (Dick and Guglielmo, 2019; Price and Guglielmo, 2009).

In terms of n-3 LCPUFAs altering membrane fluidity, we were unable in the current study to fully evaluate the potential effects on oxidative capacity. CPT is a mitochondrial membrane-bound enzyme, and its activity increases with increasing n-3 LCPUFAs in the mitochondrial membrane (Power et al., 1997). Our enzyme assay protocol disrupts the cellular membranes during the homogenization process, making it hard to assess the influence of membrane fluidity. Dietary 18:2 n-6 decreases net mitochondrial reactive oxygen species in the flight muscle in European starlings (*Sturnus vulgaris*), which could be due to alterations to membrane composition (Gerson, 2012). Furthermore, n-3 LCPUFA supplementation in humans may decrease exercise-induced muscle damage and improve recovery (Yang et al., 2023). Decreasing muscle damage and improved recovery may be beneficial to the overall migratory performance of birds by increasing flight duration and/or decreasing recovery time at stopovers between flights.

One aspect we were unable to test in the current study, but which should be considered when evaluating migratory performance, is the energetic cost of flight. Augmenting flight muscle oxidative capacity in warblers did not correlate with increased energy efficiency (Dick and Guglielmo, 2019). However, increasing flight efficiency could have many benefits including longer flights or shorter refuelling times. High intake of the n-6 PUFA 18:2 n-6 improved flight efficiency in European starlings (Carter et al., 2020; McWilliams et al., 2020), but this is likely not the result of an increase in oxidative capacity (Carter et al., 2021; Price et al., 2022). In humans, prolonged n-3 LCPUFA supplementation can decrease oxygen consumption during intense aerobic exercise, and could indicate lower energy use (Hingley et al., 2017), but the mechanism is not fully understood. Birds evolving in marine and aquatic environments that are rich in n-3 LCPUFAs may have similar improved energy use during flight. Assessing the potential of n-3 LCPUFAs should be integrative and examine multiple levels from mitochondrial and whole-animal performance. Robust studies investigating the multiple effects n-3 LCPUFAs may have on health and performance will also provide benchmarks for dietary intake requirements of n-3 LCPUFAs. We speculated that sandpipers may have low conversion rates of 18:3 n-3 to n-3 LCPUFAs based on patterns in fatty acid composition. However, we found no difference in metabolic capacity, suggesting that there was no consequence of the apparent low conversion capacity. Future studies will help to clarify this question.

In summary, we did not observe a coordinated alteration in flight muscle fatty acid metabolism or oxidation when altering the type and presence of n-3 PUFAs provided in the diet. We provided initial evidence that suggests western sandpipers may not endogenously synthesize enough EPA and DHA from 18:3 n-3 to match the proportions found in wild birds, and that a dietary source of EPA and DHA is needed to obtain the fatty acid composition observed in wild birds. Given these findings combined with global climate change potentially decreasing the natural availability of EPA/DHA (Colombo et al., 2017), future studies need to examine the importance of these fatty acids to migratory birds using a fully integrative approach including flight performance to clarify their value in the diets of sandpipers and other shorebirds.

## Supplementary Material

10.1242/jexbio.246105_sup1 Supplementary information
