# Supplementary material for: Experimental evidence that EPA and DHA are dietary requirements in a migratory shorebird, but they do not affect muscle oxidative capacity
Source: J Exp Biol. 2024 Feb 21;227(4):jeb246105. doi: 10.1242/jeb.246105 (PMC10911131; doi:10.1242/jeb.246105)
Supplement: Supplementary information [file jexbio-227-246105-s1.pdf]

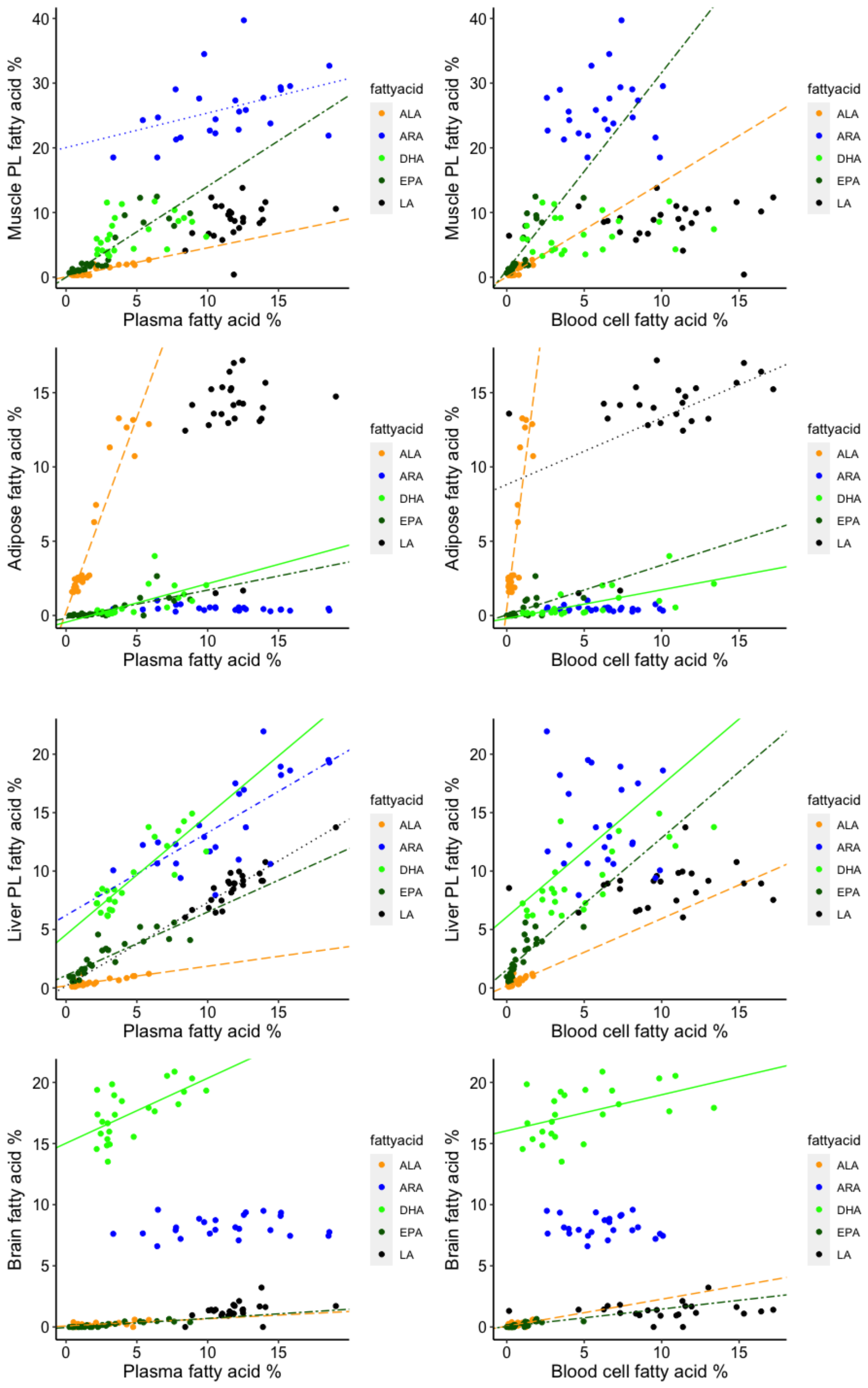

Fig. S1. Correlations of key PUFA of plasma and blood cell fatty acid composition with muscle phospholipids (PL) and adipose, liver PL and brain. Significant linear relationships ($p < 0.05$) are marked with a line of best fit. ALA: 18:3 n-3; ARA: 20:4 n-6; DHA: 22:6 n-3; EPA: 20:5 n-3, LA: 18:2 n-6.

Journal of Experimental Biology • Supplementary information

Table S1. Plasma and blood cell fatty acid composition of sandpipers fed the experimental diets.

	ALA		LCPUFA		MUFA		F- value	P - value
				Plasma fatty acid composition				
16:0	18.99	±0.94	20.15	±1.71	18.86	±0.74	$F_{2,21}$= 0.346	0.710
18:0	20.94	±1.12	18.38	±2.66	20.46	±0.86	$F_{2,21}$= 0.608	0.550
18:1 n-9	23.23	±1.82	21.15	±1.61	24.91	±1.46	$F_{2,21}$= 1.328	0.290
18:1 n-7	1.08	±0.03^{b}	1.1	±0.05^{ab}	1.25	±0.04^{a}	$F_{2,21}$= 4.131	0.031
18:2 n-6	12.81	±0.97	10.82	±0.62	12.2	±0.43	$F_{2,21}$= 2.077	0.150
18:3 n-3 (ALA)	3.83	±0.48^{a}	1.03	±0.14^{b}	0.75	±0.09^{b}	$F_{2,21}$= 33.52	<0.001
20:4 n-6 (ARA)	9.51	±1.73^{b}	10.02	±0.91^{ab}	14.08	±0.97^{a}	$F_{2,21}$= 3.933	0.035
20:5 n-3 (EPA)	2.06	±0.26^{b}	6.05	±0.64^{a}	0.8	±0.15^{b}	$F_{2,21}$= 45.6	<0.001
22:5 n-3	0.53	±0.09^{b}	1.08	±0.18^{a}	0.44	±0.13^{b}	$F_{2,21}$= 35.06	<0.001
22:6 n-3 (DHA)	3.24	±0.3^{b}	7.74	±0.47^{a}	2.78	±0.13^{b}	$F_{2,21}$= 67.38	<0.001
SFA	40.4	±0.47	38.92	±1.89	39.72	±0.96	$F_{2,21}$= 0.35	0.709
MUFA	25.34	±1.95	23.14	±1.64	27.2	±1.49	$F_{2,21}$= 1.417	0.265
PUFA	33.84	±1.86	37.84	±1.62	32.7	±1.16	$F_{2,21}$= 2.945	0.075
n-6 PUFA	22.99	±2.26	21.19	±1.39	26.86	±0.95	$F_{2,21}$=3.164	0.063
n-3 PUFA	10.69	±0.44^{b}	16.48	±0.88^{a}	5.73	±0.33^{c}	$F_{2,21}$= 80.11	<0.001
n-3 LCPUFA	6.05	±0.21^{b}	14.91	±0.89^{a}	4.11	±0.63^{b}	$F_{2,21}$= 108.8	<0.001
				Blood cell fatty acid composition				
16:0	19.54	± 2.41^{b}	19.22	±4.08^{b}	31.32	±3.16^{a}	$F_{2,21}$= 4.384	0.026
18:0	32.76	±1.57	36.23	±2.10	35.51	±1.92	$F_{2,21}$= 0.954	0.40
18:1 n-9	15.43	±0.93	13.77	±1.74	11.82	±1.19	$F_{2,21}$= 1.848	0.18
18:1 n-7	1.55	±0.14	1.53	±0.17	1.17	±0.13	$F_{2,21}$= 2.011	0.16
18:2 n-6	13.54	±0.97^{a}	8.51	±1.33^{b}	8.51	±0.98^{b}	$F_{2,21}$= 6.802	0.005
18:3 n-3 (ALA)	1.13	±0.14^{a}	0.35	±0.09^{b}	0.22	±0.03^{b}	$F_{2,21}$= 28.46	<0.001
20:4 n-6 (ARA)	6.9	±0.66	5.57	±0.87	6	±0.92	$F_{2,21}$= 0.635	0.54
20:5 n-3 (EPA)	0.73	±0.16^{b}	2.06	±0.43^{a}	0.23	±0.05^{b}	$F_{2,21}$= 12.19	<0.001
22:5 n-3	0.78	±0.10^{b}	1.35	±0.17^{a}	0.31	±0.07^{c}	$F_{2,21}$= 19.1	<0.001
22:6 n-3 (DHA)	3.94	±0.47^{b}	8.53	±1.12^{a}	2.12	±0.33^{b}	$F_{2,21}$= 20.71	<0.001
SFA	53.23	±3.4^{a}	56.15	±4.36^{ab}	67.94	±3.78^{a}	$F_{2,21}$= 4.445	0.026
MUFA	18.22	±1.15	16.44	±2.23	13.76	±1.35	$F_{2,21}$= 2.078	0.15
PUFA	28.04	±2.44	27.1	±2.5	17.98	±2.4	$F_{2,21}$= 5.608	0.011
n-6 PUFA	21.14	±1.66^{a}	14.37	±1.33^{b}	14.86	±1.93^{b}	$F_{2,21}$= 5.268	0.014
n-3 PUFA	6.9	±0.86^{b}	12.72	±1.93^{a}	3.11	±0.49^{b}	$F_{2,21}$= 17.37	<0.001
n-3 LCPUFA	5.62	±0.75^{b}	11.98	±1.64^{a}	2.69	±0.44^{b}	$F_{2,21}$= 19.64	<0.001

Values are mean ± SEM for key n-3 and n-6 PUFA, and fatty acids with a proportion of >1% in at least one group. Fatty acid means that have different letter groupings differ significantly ($p < 0.05$). SFA, saturated fatty acids; MUFA, monounsaturated fatty acids; PUFA, polyunsaturated fatty acids; LCPUFA, long-chain PUFA.

Table S2. Adipose and muscle fatty acid composition of sandpipers fed the experimental diets.

	ALA		LCPUFA		MUFA		F- value	P - value
**Adipose fatty acids composition**								
16:0	15.31	±1.10[b]	20.94	±0.62[a]	20.98	±0.74[a]	$F_{2,21}$= 14.9	<0.001
18:0	9.31	±0.50	8.24	±0.24	8.35	±0.16	$F_{2,21}$= 3.165	0.063
18:1 n-9	42.92	±1.50[b]	44.66	±2.09[b]	51.90	±1.50[a]	$F_{2,21}$= 12.94	<0.001
18:1 n-7	1.25	±0.04	1.15	±0.05	1.02	±0.22	$F_{2,21}$= 0.758	0.48
18:2 n-6	15.83	±0.32[a]	12.74	±0.34[ab]	10.55	±1.96[b]	$F_{2,21}$= 5.218	0.015
18:3 n-3 (ALA)	10.97	±0.96[a]	2.45	±0.26[b]	1.91	±0.11[b]	$F_{2,21}$= 82.77	<0.001
20:4 n-6 (ARA)	0.43	±0.03[b]	0.61	±0.08a	0.37	±0.01[b]	$F_{2,21}$= 7.26	0.004
20:5 n-3 (EPA)	0.06	±0.02[b]	1.04	±0.27[a]	0.031	±0.01[b]	$F_{2,21}$= 13.42	<0.001
22:6 n-3 (DHA)	0.26	±0.032[b]	1.8	±0.37[a]	0.16	±0.02[b]	$F_{2,21}$= 17.7	<0.001
SFA	25.23	±0.65[b]	30.01	±0.76[a]	30.3	±0.83[b]	$F_{2,21}$= 14.46	<0.001
MUFA	46.64	±1.57[b]	49.09	±0.87[b]	56.3	±1.4[a]	$F_{2,21}$= 14.66	<0.001
PUFA	27.96	±0.97[a]	20.75	±0.83[b]	13.26	±1.9[c]	$F_{2,21}$= 30.93	<0.001
n-6 PUFA	16.37	±0.33[a]	14.48	±0.32[ab]	10.97	±1.95[b]	$F_{2,21}$= 5.569	0.015
n-3 PUFA	11.6	±0.99[a]	6.26	±0.8[b]	2.28	±0.15[c]	$F_{2,21}$= 39.81	<0.001
n-3 LCPUFA	0.52	±0.08[b]	3.75	±0.74[a]	0.26	±0.06[b]	$F_{2,21}$= 20.48	<0.001
**Flight muscle phospholipid fatty acid composition**								
16:0	6.91	±1.57	7.35	±0.75	7.14	±1.16	$F_{2,21}$= 0.032	0.97
18:0	36.33	±0.58	36.46	±0.60	35.52	±0.77	$F_{2,21}$= 0.593	0.56
18:1 n-9	5.65	±0.80[b]	6.18	±0.28[ab]	7.75	±0.40[b]	$F_{2,21}$= 4.068	0.032
18:1 n-7	1.86	±0.72	1.24	±0.03	1.45	±0.06	$F_{2,21}$= 0.583	0.57
18:2 n-6	9.73	±1.43[b]	6.59	±0.45[a]	9.68	±0.36[b]	$F_{2,21}$= 4.047	0.033
18:3 n-3 (ALA)	1.93	±0.14[a]	0.34	±0.01[b]	0.4	±0.01[b]	$F_{2,21}$= 119.5	<0.001
20:4 n-6 (ARA)	26.45	±2.21[ab]	22.16	±0.64[b]	29.46	±1.02[a]	$F_{2,21}$= 17.7	<0.001
20:5 n-3 (EPA)	1.88	±0.16[b]	9.47	±0.75[a]	1.14	±0.16[b]	$F_{2,21}$= 103.7	<0.001
22:5 n-3	0.92	±0.16[ab]	1.10	±0.07[a]	0.57	±0.03[b]	$F_{2,21}$= 7.462	0.004
22:6 n-3 (DHA)	6.87	±1.18[ab]	8.31	±0.82[a]	4.99	±0.56[b]	$F_{2,21}$= 3.485	0.049
SFA	43.58	±1.72	44.05	±0.38	43.37	±0.74	$F_{2,21}$= 0.099	0.91
MUFA	7.93	±0.41[b]	7.67	±0.29[b]	9.65	±0.43[a]	$F_{2,21}$= 7.981	0.003
PUFA	48.29	±1.87	48.23	±0.43	45.4	±1.72	$F_{2,21}$= 1.231	0.31
n-6 PUFA	36.43	±1.04[a]	28.82	±1.06[b]	37.98	±1.73[a]	$F_{2,21}$= 13.93	<0.001
n-3 PUFA	11.86	±1.28[b]	19.41	±1.32[a]	7.41	±0.53[c]	$F_{2,21}$= 30.12	<0.001
n-3 LCPUFA	9.72	±1.31[b]	18.90	±1.32[a]	6.80	±0.53[b]	$F_{2,21}$= 31.93	<0.001

Values are mean ± SEM for key n-3 and n-6 PUFA, and fatty acids with a proportion of >1% in at least one group. Fatty acid means that have different letter groupings differ significantly (p < 0.05). SFA, saturated fatty acids; MUFA, monounsaturated fatty acids; PUFA, polyunsaturated fatty acids; LCPUFA, long-chain PUFA.

Journal of Experimental Biology • Supplementary information

Table S3. Fatty acid composition of the liver phospholipids and brain of western sandpipers fed the experimental diets.

	ALA		LCPUFA		MUFA		F- value	P – value
**Liver phospholipid fatty acid composition**								
16:0	15.35	±1.38[b]	19.41	±0.89[a]	18.06	±0.85[ab]	$F_{2,21}$= 3.747	0.04
18:0	31.22	±0.98	27.92	±1.11	30.02	±0.90	$F_{2,21}$= 2.782	0.08
18:1 n-9	13.93	±1.34	13.11	±1.47	13.20	±0.83	$F_{2,21}$= 0.0128	0.88
18:1 n-7	0.96	±0.3	0.95	±0.07	1.03	±0.05	$F_{2,21}$= 0.746	0.48
18:2 n-6	9.69	±0.68	7.86	±0.54	8.53	±0.36	$F_{2,21}$= 2.919	0.070
18:3 n-3 (ALA)	0.81	±0.10[a]	0.47	±0.12[ab]	0.26	±0.04[b]	$F_{2,21}$= 8.61	0.002
20:4 n-6 (ARA)	13.9	±1.06[b]	10.5	±0.46[c]	18.0	±0.91[a]	$F_{2,21}$= 19.59	<0.001
20:5 n-3 (EPA)	2.74	±0.37[b]	4.39	±0.38[a]	1.03	±0.16[c]	$F_{2,21}$= 25.76	<0.001
22:6 n-3 (DHA)	8.01	±0.34[b]	12.85	±0.59[a]	6.86	±0.29[b]	$F_{2,21}$= 55.25	<0.001
SFA	46.83	±0.92	47.6	±1.51	48.46	±0.58	$F_{2,21}$= 0.56	0.580
MUFA	15.36	±1.41	14.67	±1.65	14.73	±0.91	$F_{2,21}$= 0.081	0.922
PUFA	37.35	±1.18	37.55	±0.92	36.37	±0.75	$F_{2,21}$= 0.425	0.659
n-6 PUFA	24.48	±1.27[a]	18.7	±0.46[b]	27.19	±0.78[a]	$F_{2,21}$= 23.12	<0.001
n-3 PUFA	12.73	±0.46[b]	18.71	±0.67[a]	9.05	±0.42[c]	$F_{2,21}$= 85.75	<0.001
n-3 LCPUFA	11.63	±0.41[b]	18.04	±0.68[a]	8.49	±0.41[c]	$F_{2,21}$= 89.64	<0.001
**Brain fatty acid composition**								
16:0	28.50	±1.77	26.49	±2.17	28.60	±1.27	$F_{2,21}$= 0.451	0.64
18:0	24.11	±0.69	23.85	±0.89	23.87	±0.86	$F_{2,21}$= 0.033	0.97
18:1 n-9	11.80	±0.26	12.48	±0.79	13.30	±0.81	$F_{2,21}$= 1.263	0.30
18:1 n-7	5.12	±0.20	5.18	±0.14	5.49	±0.25	$F_{2,21}$= 0.954	0.40
18:2 n-6	1.33	±0.09	1.29	±0.23	1.35	±0.32	$F_{2,21}$= 0.016	0.92
18:3 n-3 (ALA)	0.31	±0.07[a]	0.16	±0.02[ab]	0.11	±0.05[b]	$F_{2,21}$= 4.051	0.033
20:4 n-6 (ARA)	8.45	±0.31	7.66	±0.24	8.49	±0.27	$F_{2,21}$= 2.905	0.08
20:5 n-3 (EPA)	0.08	±0.03[b]	0.44	±0.04[a]	0.004	±0.004[b]	$F_{2,21}$= 57.45	<0.001
22:6 n-3 (DHA)	17.11	±0.64[b]	19.26	±0.44[a]	16.06	±0.66[b]	$F_{2,21}$= 7.657	0.003
SFA	53.81	±1.09	51.18	±1.58	53.21	±1.08	$F_{2,21}$= 1.168	0.33
MUFA	17.54	±0.32	18.53	±1.08	19.55	±0.92	$F_{2,21}$= 1.431	0.26
PUFA	28.34	±1.00[b]	30.03	±0.84[a]	26.83	±0.70[b]	$F_{2,21}$= 11.95	<0.001
n-6 PUFA	10.10	±0.34	9.24	±0.43	10.13	±0.35	$F_{2,21}$= 1.802	0.19
n-3 PUFA	18.24	±0.68[b]	20.78	±0.46[a]	16.70	±0.64[b]	$F_{2,21}$= 11.66	<0.001
n-3 LCPUFA	17.83	±0.65[b]	20.57	±0.45[a]	16.53	±0.67[b]	$F_{2,21}$= 11.95	<0.001

Values are mean ± SEM for key n-3 and n-6 PUFA, and fatty acids with a proportion of >1% in at least one group. Fatty acid means that have different letter groupings differ significantly ($p < 0.05$). SFA, saturated fatty acids; MUFA, monounsaturated fatty acids; PUFA, polyunsaturated fatty acids; LCPUFA, long-chain PUFA.

Journal of Experimental Biology • Supplementary information

Table S4. PCA loadings of the CLR transformed fatty acids.

	PC1	PC2	PC3	PC4
16:0	0.0619	-0.4971	0.0239	-0.4609
18:0	-0.2928	-0.3093	-0.5171	0.3593
18:1 n-9	0.2946	-0.4317	0.1160	-0.0620
18:1 n-7	-0.2124	-0.3973	0.3267	0.1045
18:2 n-6	0.3737	0.0188	-0.6419	-0.2776
18:3 n-3	0.4347	-0.0021	0.1812	0.6723
20:4 n-6, ARA	-0.4624	0.0958	-0.3154	0.1984
20:5 n-3, EPA	-0.0238	0.5463	0.1624	-0.2308
22:6 n-3, DHA	-0.4853	-0.0705	0.2030	-0.1483
Standard deviation	1.8885	1.7281	0.9031	0.7667
Proportion of Variance	0.3963	0.3318	0.0906	0.0653
Cumulative Proportion	0.3963	0.7281	0.8187	0.8840

Table S5. Results of the linear regression of key n-3 and n-6 PUFA of plasma and blood cell fatty acid composition with tissue fatty acid composition

	Plasma				Blood Cell			
	intercept	slope	R^2	P - value	intercept	slope	R^2	P - value
**Liver**								
18:2 n-6	0.174	0.713	0.86	<0.001	7.743	0.093	0.04	0.307
20:4 n-6	6.212	0.707	0.51	<0.001	15.658	-0.246	0.02	0.517
18:3 n-3	0.184	0.168	0.57	<0.001	0.173	0.576	0.59	<0.001
EPA	1.076	0.543	0.71	<0.001	1.550	1.129	0.55	<0.001
DHA	4.567	1.019	0.74	<0.001	6.076	0.651	0.56	<0.001
**Flight Muscle**								
18:2 n-6	3.346	0.445	0.07	0.117	7.684	0.096	0.02	0.542
20:4 n-6	20.040	0.534	0.15	0.037	24.606	0.229	0.02	0.640
18:3 n-3	0.055	0.449	0.85	<0.001	0.074	1.453	0.79	<0.001
EPA	-0.011	1.406	0.77	<0.001	1.091	3.053	0.65	<0.001
DHA	4.640	0.454	0.12	0.523	5.919	0.166	0.04	0.346
**Brain**								
18:2 n-6	-0.041	0.114	0.10	0.069	1.196	0.013	0.02	0.722
20:4 n-6	7.827	0.033	0.02	0.468	8.531	-0.054	0.02	0.512
18:3 n-3	0.079	0.059	0.32	0.002	0.065	0.221	0.40	<0.001
EPA	-0.047	0.075	0.81	<0.001	0.031	0.143	0.52	0.004
DHA	15.025	0.532	0.37	<0.001	16.042	0.295	0.19	<0.001
**Adipose**								
18:2 n-6	10.822	0.214	0.03	0.588	8.815	0.448	016	0.028
20:4 n-6	0.654	-0.016	0.12	0.057	0.535	-0.011	0.02	0.519
18:3 n-3	0.226	2.619	0.90	<0.001	0.472	8.237	0.78	<0.001
EPA	-0.186	0.190	0.55	<0.001	0.040	0.335	0.29	0.004
DHA	-0.452	0.260	0.41	<0.001	-0.199	0.193	0.44	<0.001

Journal of Experimental Biology • Supplementary information
